# Factors Influencing the Screw Stability of Implant-Supported Single Crowns: An In Vitro Study

**DOI:** 10.3390/ma18030506

**Published:** 2025-01-23

**Authors:** Shaza Bishti, Mohamad Shams Eddin Alsagher, Martin Homa, Stefan Wolfart, Taşkın Tuna

**Affiliations:** Department of Prosthodontics and Biomaterials, Center for Implantology, Uniklinik RWTH Aachen, 52074 Aachen, Germany

**Keywords:** implant-supported, crown, screw loosening, sealant, disinfectant, cement-retained, screw-retained

## Abstract

The aim is to investigate the impact of retention type, implant/abutment angulation, and the presence of sealant/antimicrobial agents on screw loosening of implant-supported restorations. Fifty dental implants along with their respective abutments and screws were allocated to five groups (n = 10). The groups were categorized based on type of crown retention (screw-/cement-retained), implant/abutment angulation (0°/20°), and type of disinfectant/sealant as follows: Cem_control (cemented/0°/none), Cem_GP (cemented/0°/gutta-percha), Cem_CHX (cemented/0°/chlorhexidine), Cem_Ang (cemented/20°/none), and Screw (screwed/0°/ none). Abutment screws were tightened (20 Ncm), and CAD/CAM zirconia crowns were fabricated. Glass ionomer cement was used for crown cementation in the cemented groups. Samples were subjected to dynamic loading in a chewing simulator (1,200,000 cycles/98 N). After loading, the reverse torque values (RTVs) of the abutment screws were determined (Ncm) using an electronic screwdriver, and the reverse torque difference (RTD) was subsequently calculated. The lowest RTD was reported in group Cem_GP (−2.22 ± 1.03), whereas the highest RTD was seen in group Screw (−4.65 ± 1.79). Group Screw showed a statistically significant difference from all other groups (*p* < 0.05). No statistically significant difference between the cemented test groups Cem_GP, Cem_CHX, and Cem_Ang and the control group was found. Screw-retained restorations exhibited significantly greater RTD values compared to cement-retained ones. Implant/abutment angulation and the sealant/disinfectant appeared to have no notable effect on the screw stability of single-implant restorations.

## 1. Introduction

Dental implants have become a widely recognized and reliable approach for rehabilitating partially edentulous patients, offering predictable long-term outcomes [1,2]. Implant-supported restorations used to restore single-tooth gaps have demonstrated high long-term survival rates [3,4,5,6,7,8]. According to a meta-analysis, a survival of 93% and 94.4% was reported for implant-supported single crowns (SCs) after 5 and 10 years of clinical service, respectively [7]. 

However, despite their favorable outcomes, implant-supported restorations are subject to biological and technical complications [9,10]. The most common technical problems with implant-supported restorations are screw loosening, screw fracture, fracturing of veneering porcelain, and framework fracture [9,11].

Abutment screw loosening is the most common and challenging prosthetic complication of implant-supported restorations [12,13], ranging between 3% and 45%. Implant-supported fixed dental prostheses (FDPs) revealed 5.3% screw loosening after 5 years [14]. In a further meta-analysis, an estimated rate of 3.6% and 5.2% for screw or abutment loosening in SCs after 5 and 10 years was reported [7,9]. These rates are lower than the cumulative percentage of 8.8% reported by Jung et al. 2012 and the 5.1% after 5 years documented by Sailer et al. 2009 [9,15]. However, in those reviews, the high incidence of screw loosening was primarily attributed to the older gold screw designs used in two studies [16,17]. Advances in screw designs and materials seem to reduce this technical issue, although they do not completely eliminate it.

Maintenance of a stable implant–abutment connection is critical for the long-term success of single-tooth implant restorations [18]. Several investigations have been conducted to examine the factors that may influence implant–abutment screw interface stability. According to several studies, the type of abutment screw material seems to play a role in the incidence of screw loosening [19,20,21,22]. Moreover, different types of fabrication methods for implant abutments (stock abutment, gold cast abutment, and CAD/CAM custom abutment) affected the initial screw loosening [23]. On the other hand, different implant–abutment connection geometries such as external and internal connections did not induce screw loosening, provided that proper anti-rotational features and torque were employed [24]. In contrast, a systematic review evaluating the incidence of screw loosening in external and internal connection metal- or zirconia-based abutments in single-implant restorations reported a higher frequency of screw loosening in externally connected implant systems for both types of materials [12]. Occlusion is another possible factor that has been discussed in the literature for its influence on screw stability [24,25,26,27]. Restoration design, occlusal table width, and occlusal force distribution are crucial factors that should be considered to minimize the risk of screw loosening and ensure the longevity of implant-supported restorations [28]. Although studying implant occlusion is challenging, since the magnitude and the direction of the occlusal force are difficult to calculate, several recommendations have been mentioned in the literature to decrease occlusal overload and consequently reduce the risk of screw loosening [27]. These include increasing contact points, monitoring parafunctional habits, narrowing the occlusal table, and reducing cantilevers [27].

Fixed implant-supported restorations can be either screw- or cement-retained. The outcome and complications of these restorations in partially edentulous patients have been previously investigated [11]. Here, cemented restorations showed better long-term clinical and biological outcomes compared to screw-retained ones. Abutment screw loosening was statistically more frequent in screw-retained fixed dental prostheses (32 ± 0.3%) than in cement-retained ones (9 ± 0.2%). The greater preload exerted by the reduced passive fit of the screw-retained framework was suggested to be a possible reason for the greater frequency of abutment screw loosening in fixed dental prostheses [11]. In this context, it is noteworthy to mention that the lack of a standardized reporting protocol for prosthodontic maintenance and complications of fixed implant-supported restorations makes it difficult to compare the prosthodontic outcome between screw- and cement-retained implant restorations [29].

One of the main difficulties in implant-supported restorations is preventing microbial penetration into the internal parts of the implant through the implant–abutment connection [30,31]. Various efforts have been made to minimize internal bacterial colonization. These include the use of antimicrobial agents such as chlorhexidine solutions or sealing the interface using silicone materials [32,33,34]. Several studies have reported the efficiency of these agents in reducing bacterial colonization in the implant–abutment junction [32,35]. However, the effect of these agents on the screw stability of implant restorations has been rarely reported in the literature. One in vitro study investigated the influence of chlorohexidine gel and a silicone sealant on the reverse torque values of implant single crowns after chewing simulation representing 2 years of function [36]. Here, the application of chlorohexidine as an antimicrobial agent did not significantly affect the implant–abutment connection stability under cyclic loading, whereas the silicone sealant caused lower reverse torque values under functional loads [36].

Given the critical role of screw stability in the long-term success of implant-supported restorations, this in vitro study seeks to investigate the key factors influencing screw retention in single-implant restorations, with a focus on retention type, implant angulation, and the role of disinfectants and sealants in maintaining screw stability. The three null hypotheses were as follows: (1) the type of retention, (2) implant/abutment angulation, and (3) the presence of antimicrobial/sealant agents have no influence on the screw stability of single-implant-supported restorations.

## 2. Materials and Methods

### 2.1. Study Design

**Figure 1:**
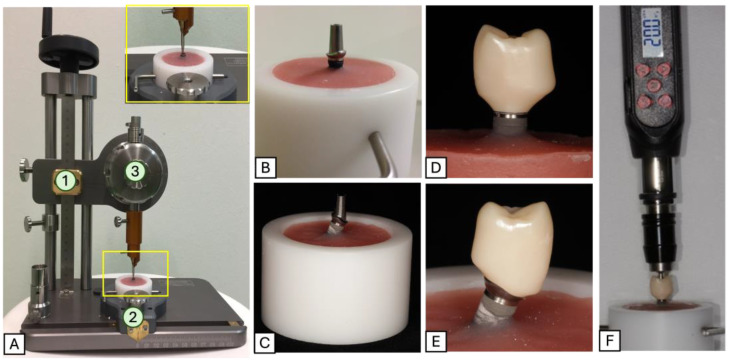
(**A**) Holding device with adjustable alignment across three axes (1: horizontal, 2: vertical, 3: angulation) used for embedding the implants in the holders in a standardized manner; (**B**,**C**) embedded straight and angulated implant in the sample holder filled with acrylic resin (implant shoulder 2 mm above resin level); (**D**,**E**) tested crown placed onto its corresponding straight/angulated implant abutment. (**F**) A sample screwed to its implant using an electronic screwdriver to a torque of 20 Ncm.

Fifty dental implants (Promote Plus, CAMLOG Biotechnologies AG, Basel, Switzerland) with a diameter of 4.3 mm and a length of 13 mm were embedded in a standardized manner with the aid of a holding device in plastic cylindrical sample holder filled with autopolymerizing acrylic resin (PalaXpress, Kulzer GmbH, Hanau, Germany) (Figure 1). The implant shoulder was placed 2 mm above the resin level to simulate marginal bone loss of bone-level implants (DIN ISO 14801) [37]. The prepared samples were allocated to 5 groups (n = 10/group) according to the type of retention, implant/abutment angulation, and the presence or absence of sealant/disinfectant (Table 1).

Implants in groups Cem_control, Cem_GP, and Cem_CHX were straight and provided with straight titanium abutments (Esthomic Abutment, gingival height 1–1.8 mm, Camlog Biotechnologies AG, Basel, Switzerland), Group Cem_Ang received 20° angulated implants and were provided with 20° angulated titanium abutments (Esthomic Abutment 20°, gingival height 1–1.8 mm, Type A, Camlog Biotechnologies AG, Basel, Switzerland), and group Screw was provided with straight implants and CAD/CAM titanium abutments (Titanium base CAD/CAM abutment, Camlog Biotechnologies AG, Basel, Switzerland). CAD/CAM monolithic zirconia crowns (Zirlux FC2, Henry Schein, Langen, Germany) were fabricated and cemented in a standardized manner to the abutments in groups Cem_control, Cem_GP, Cem_CHX, and Cem_Ang using glass ionomer cement (Ketac Cem Aplicap, 3M Espe, Seefeld, Germany). Group Screw received screw-retained monolithic zirconia crowns.

**Figure 2:**
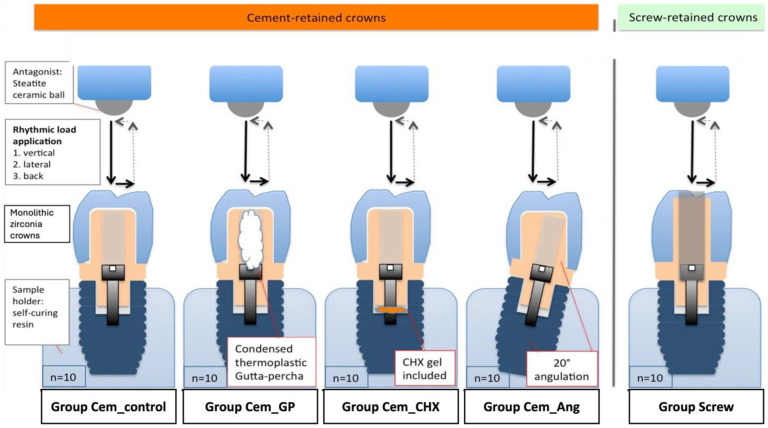
A: Schematic image showing the different study groups. Group Cem_control (cemented, straight abutment, empty screw chambers), group Cem_GP (cemented, straight abutment, gutta-percha in the screw chamber of the abutment), group Cem_CHX (cemented, straight abutment, chlorohexidine gel in the screw chamber of the implant), group Cem_Ang (cemented, 20° angulated abutment, empty screw chambers), and group Screw (screwed, straight abutment, empty screw chambers). Arrows between the implant crowns and antagonists represent the load in the vertical and lateral direction.

Screw chambers of the implants were left empty in groups Cem_control, Cem_ang, and Screw. Group Cem_GP was filled with a thermoplastic gutta-percha (Gutta-Percha, VDW GmbH, Munich, Germany) in the screw access hole above the screw, whereas group Cem_CHX was provided with 1% chlorohexidine gel (Chlorhexamed, Haleon Germany GmbH, Munich, Germany). The CHX gel was injected into the screw access holes of the implant fixtures until they were filled; then, the screw was fixed and tightened in place. Details of the tested groups and the materials used are presented in Figure 2.

### 2.2. Sample Preparation

Abutments of the cemented groups (Cem_control, Cem_GP, Cem_CHX, and Cem_Ang) were screwed to their corresponding implants according to the manufacturer’s instructions with a torque of 20 Ncm using an electronic screwdriver (200 CNM, Holex, Hoffmann Group, Munich, Germany). To avoid the settling effect, retightening with the same torque value was applied after 10 min by the same operator.

Afterward, the inner surface of each crown was air-abraded using 50 μm aluminum oxide at a pressure of 1 bar for 15 s at a distance of 10 mm. All crowns were cleaned in 70% alcohol using an ultrasonic device (Renfert Easyclean, Hilzingen, Germany) for 5 min and then dried with air pressure.

For the cemented groups (Cem_control, Cem_GP, Cem_CHX, and Cem_Ang), glass ionomer cement (Ketac Cem Aplicap, 3M Espe, Seefeld, Germany) was mixed for 10 s using a centrifugal capsule mixing unit (Espe Roto Mix, 3M Espe, Seefeld, Germany) and then applied in a thin layer to the intaglio surface of the crowns. The crowns were then placed on the abutments with finger pressure and mounted under a pressure of 10 N with special clamps for 10 min at room temperature until the cement was completely set.

**Figure 3:**
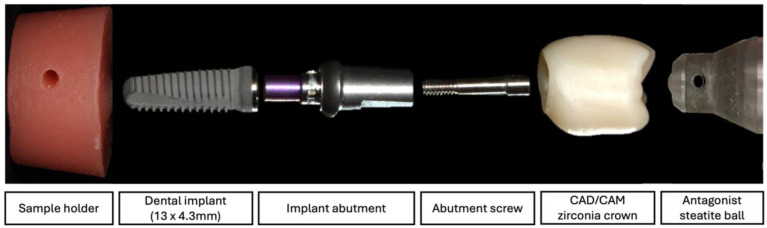
Different parts of the investigated cemented straight implant restoration samples.

For group Screw, the crowns were luted to their corresponding abutments (Titanium base CAD/CAM abutment, Camlog Biotechnologies AG, Switzerland) using a self-curing luting composite (Multilink Hybrid Abutment cement, Ivoclar Vivadent, Schaan, Liechtenstein). Restorations were then screwed to their implants according to the manufacturer’s instructions with a torque of 20 Ncm, then retightened after 10 min. The screw access channel was then closed with a foam pellet and composite material (Tetric EvoCeram, Ivoclar Vivadent, Pforzheim, Germany). The different parts of the investigated cemented implant restoration samples are shown in Figure 3.

### 2.3. Dynamic Loading Tests

**Figure 4:**
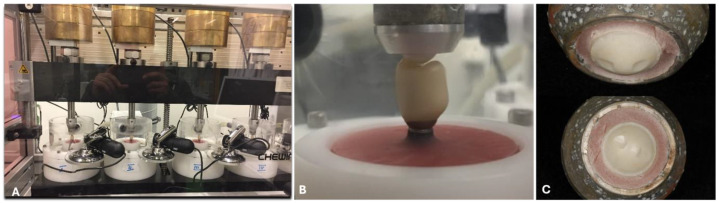
(**A**) Tested samples loaded into the universal testing machine; (**B**) a closer image of the investigated implant crown with its antagonist; (**C**) steatite balls after loading showing two wear facets due to the excessive lateral forces.

All samples were placed in a dual-axis chewing simulator (CS-4.8, SD Mechatronik, Feldkirchen, Westerham, Germany) with 8 hosted samples at a time. Steatite ceramic balls with a diameter of 6 mm were used as antagonists and were fixed to the upper sample holders to transfer vertical and lateral load to the implant-restoration samples. Samples were then subjected to thermocycling (6336 cycles, 5–55 °C; dwell time = 60 s) and a chewing load of 98 N for 1.2 million cycles, simulating a clinical service time of 5 years in the oral cavity (Figure 4).

### 2.4. Reverse Torque Measurements

After artificial aging, an occlusal opening in the crowns of the cemented groups was drilled without pressure under water cooling to access the screw access channels. The foam pellet was removed. For the screwed group, the composite filling and the foam pellet were removed. Samples were fixed, and the torque strength needed to remove the abutment screw (reverse torque value (RTV)) was measured using an electronic screwdriver (200 CNM, Holex, Germany). Finally, the reverse torque differences (RTD) for each sample were calculated as follows: RTD (Ncm) = Preloading screw-in torque − postloading screw-out torque (RTV).

### 2.5. Statistical Analyses

The data were collected and statistically analyzed using SPSS, version 22.0.0.1 (SPSS Inc, Chicago, IL, USA). Screw stability in this study was presented as reverse torque differences (RTD) and was expressed in mean values with standard deviations. One-way ANOVA followed by the post hoc Tukey’s test was used to identify differences between the study groups. A statistical significance level of *p* < 0.05 was set.

## 3. Results

The mean values and standard deviations of reverse torque differences of the five investigated groups are presented in Table 2.

**Figure 5:**
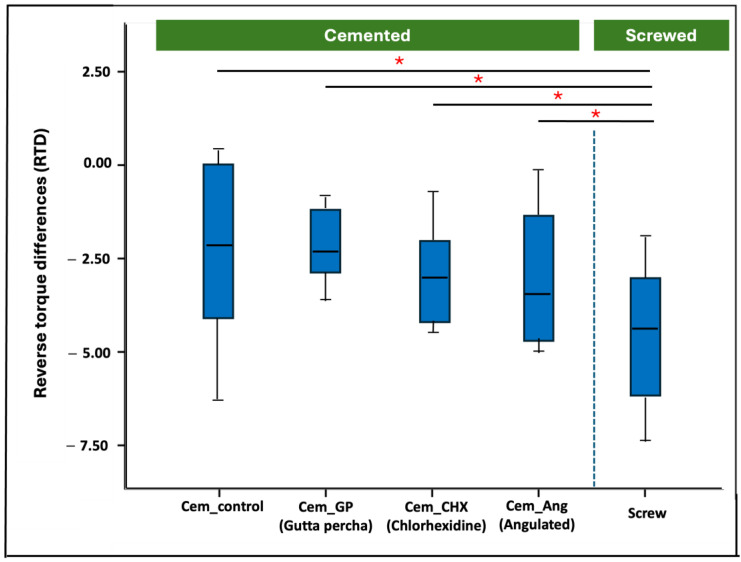
Boxplots representing the reverse torque differences within the tested groups. (*) represents significant differences between the groups (*p* < 0.05).

In general, all restorations survived the 1.2 million cycles of artificial aging without any bending or fracture or obvious screw loosening. All restorations showed a degree of reverse torque loss after artificial loading. The highest reverse torque value (RTV) was reported in group Cem_GP (17.9 ± 1.04 Ncm) with an RTD of 2.22 ± 1.03 Ncm, whereas the least RTV was seen in group Screw (15.46 ± 1.77) with an RTD of 4.65 ± 1.76 Ncm. Group Screw revealed a statistically significant difference from all other investigated groups (*p* < 0.05). On the other hand, the cemented groups (Cem_GP, Cem_CHX, and Cem_Ang) revealed no statistically significant difference from the control group (Group Cem_control) (Figure 5).

The presence of disinfectant and/or sealants in the screw access holes of implant-supported restorations showed no significant effect on the amount of reverse torque. The type of retention (screw/cemented) negatively affected the screw stability of implant-supported crowns.

## 4. Discussion

This in vitro study investigated the influence of retention type, implant/abutment angulation, and the presence of antimicrobial or sealant agents in the screw chambers on the screw stability of single-implant-supported restorations after 5 years of chewing simulation. Although clinically significant results can only be established through in vivo studies, standardized laboratory testing of implant–abutment connections may provide an understanding of the mechanical performance of the implant restorations [38].

The **type of retention**, whether screw- or cement-retained, seems to be a critical factor affecting screw stability. The results of our in vitro investigation showed statistically significant higher RTD values with screw-retained restorations in comparison to cement-retained ones. Therefore, the hypothesis that the retention type has no influence on the screw stability of single-implant-supported restorations was rejected. Here, a removal torque loss of 11.22% and 23.12% was reported for cement- and screw-retained restorations, respectively. These results align with previous studies reporting that screw-retained restorations are more prone to screw loosening than cement-retained ones due to the mechanical forces acting on the abutment screw during occlusion and function [26,39,40,41,42]. Screw loosening in implant restorations has been widely discussed in the literature, and studies have reported that this complication is commonly associated with factors such as inadequate torque application, cyclic loading, and micromotion at the implant–abutment interface [21,24,42]. Differences in the incidence of screw loosening between screw- and cement-retained restorations can be related to several biomechanical factors. Load distribution and stress absorption are critical factors for the stability of the implant–abutment connection. In cement-retained restorations, the cement layer between the restoration and the abutment acts as a cushion, which may absorb and distribute occlusal forces more evenly across the restoration–abutment–implant interface. This even distribution of forces minimizes stress concentration at the screw joint, and in turn, reduces the possibility of micromovements that can lead to screw loosening. Moreover, cement provides a bonded interface between the restoration and abutment by filling any microscopic gaps, creating a more stable connection. This type of connection can reduce micromovements at the implant–abutment interface under functional loading [43,44]. On the other hand, screw-retained restorations have a direct mechanical connection between the crown and the abutment, meaning occlusal forces can directly act on the screw. Over time, repeated loading, especially lateral forces, can lead to micromovements increasing the likelihood of screw loosening in these restorations [43,44,45].

Given its widespread clinical application in implant dentistry and its advantageous properties, particularly its semipermanent nature [46,47], glass ionomer cement was used for the cemented restorations investigated in this study. Additionally, the cartridge system of this cement facilitates optimal mixing and ensures consistent application. It is worth noting that the use of alternative cement types may yield different reverse torque values, which could be explored in future research.

**Abutment angulation** is another critical factor that may influence the screw stability of implant-supported restorations. Our findings reported higher RTD values of investigated samples with angulated abutments, with a removal torque loss of 15.13% in comparison to 11.22% for those with straight abutments. However, these differences were statistically non-significant, which may suggest limited evidence for the influence of implant/abutment angulation on the screw stability of single-implant-supported restorations under the conditions tested. In this context, previous studies have suggested that non-axial loading due to angulated abutments can provoke stresses on the implant–abutment interface, which can lead to micromovements and eventual screw loosening or failure [48,49]. Angulated abutments are usually used to correct divergences between the surgical implant axis and the optimal prosthetic alignment of the final restoration [50]. In a recent systematic review, the effect of angulated abutments, angulated screw channel abutments, and angulated implants on prosthetic screw loosening was assessed [51]. This review included studies with prosthetic angle corrections ranging between 0 and 30 degrees, and most of the studies reported an increase in screw loosening with increased abutment angle correction. Other studies found no significant differences in screw loosening due to angulation after dynamic loading [51]. Angulated abutments are subjected to greater torsional forces during mastication, which can lead to screw loosening and compromised prosthetic function. These stresses are further amplified when the screw is under suboptimal preload, as angulation affects the distribution of forces within the implant–abutment screw complex [49].

Our investigation on the application of **sealants and/or antimicrobial agents** highlighted another factor that may influence screw stability. As screw access channels in implant restorations provide a pathway for microbial contamination, the use of sealants and/or antimicrobial agents in the implant/abutment screw channel has been shown to reduce microleakage and bacterial colonization. However, these agents are factors known to compromise the stability of the abutment screw connection over time [52]. In the current study, the use of gutta-percha as a sealant revealed less removal torque loss than cemented restorations with empty screw channels. On the other hand, the presence of chlorhexidine gel in the implant below the screw increased the amount of screw loosening when compared to the control group. However, our findings did not reveal statistically significant differences, which means that the hypothesis that the presence of sealants or antimicrobial agents has no influence on the screw stability of single-implant-supported restorations could not be rejected. In contrast to our study, an in vitro study investigating the effect of chlorhexidine gel on torque and detorque values of implant abutment screws, reported high levels of screw loosening [53]. This was explained by the decrease in the coefficient of friction when lubricant pastes such as gel were used. This decrease reduced the effective preload values and resulted, therefore, in abutment screw loosening [53]. On the other hand, another study reported that the use of 0.2% liquid CHX presented satisfying outcomes in avoiding screw loosening [54]. According to a recent systematic review evaluating the influence of different sealant agents (adhesives, saliva, chlorhexidine, Vaseline, silicone gel, Polytetrafluoroethylene (PTFE) tape, normal saline) on the reverse torque values of implant restorations after cyclic loading, there is no definitive evidence that any particular agent is more effective in reducing screw loosening [54].

From a mechanical perspective, the findings of the current study indicate that the application of chlorhexidine does not significantly influence screw loosening in implant-supported restorations. Therefore, the decision to incorporate chlorhexidine should be guided by its potential biological benefits, such as its antimicrobial properties and ability to reduce microbial penetration to the implant–abutment junction, adapted to the individual patient’s clinical needs.

Nevertheless, it is noteworthy to mention that oral conditions may result in different values than those obtained in an in vitro study setting. The presence of saliva, differences in salivary density and viscosity, as well as blood contamination may affect preload and consequently influence the risk of screw loosening [55,56,57]. According to the available literature, the influence of fluid contamination on the reverse torque values of implant restorations is controversial. An in vitro study by Gumus et al. investigated the influence of fluid contamination of the implant abutment screw hole on the RTV. Here, no significant differences between the control group which had no contamination with saliva and the CHX group could be reported [58]. In another study, RTVs were significantly reduced by saliva contamination in comparison to implant samples provided with blood or CHX [55]. The controversy among studies regarding screw loosening may be attributed to the heterogeneity in experimental conditions, including screw design and materials, type of fluid contamination and/or lubrication, the concentration of CHX gel, and environmental factors such as variations in exposure to thermocycling and/or dynamic loading.

In general, when considering unscrewing, it is important to understand the mechanics of the prosthetic screw. This screw is designed to connect two components—the implant and the prosthetic abutment—by applying torque to achieve what is known as preload in the implant–abutment complex. When tightened, the screw stretches slightly, creating tension; its elastic recovery then holds the implant and abutment together with a clamping force [45]. It has been reported that around 50% of the applied torque is utilized to counteract friction between the screw head and the seating surface of the abutment, 40% to address friction along the threads, and the remaining 10% directly tightens the screw [20].

Additionally, the settling effect, which impacts screw stability, arises due to microroughness on the screw and inner implant surfaces. Because of this roughness, the components do not achieve full contact initially. As rough areas gradually flatten out, settling occurs, leading to a slight preload loss. Studies indicate that around 2 to 10% of preload is lost due to settling, influenced by surface roughness, hardness, and the torque applied, and was observed 2 to 3 min after tightening even without external forces [21,45,59]. Thread friction is greater during initial screw tightening and decreases with subsequent retightening, leading to a lower removal torque value (RTV) compared to the torque required for initial tightening [21,45,59].

Thermocycling, which simulates the temperature fluctuations in the oral environment, is usually used to assess the impact of daily temperature changes on the integrity of the implant–abutment connection [60]. However, studies have reported that thermocycling alone may not significantly affect screw loosening [60,61]. In the current study, a combination of thermocycling and mechanical loading was employed to simulate the oral environment as realistically as possible. This combined approach aimed to replicate the thermal fluctuations and masticatory forces experienced in vivo, providing a comprehensive assessment of their impact on the stability of implant–abutment connections, specifically screw stability.

Understanding the mechanics of screw tightening and the factors influencing screw stability, such as preload, frictional forces, and the settling effect, is essential to the clinical implications of this study. This investigation explores how variations in retention type, implant/abutment angulation, and the use of sealants or antimicrobial agents affect screw loosening in implant restorations. Although all investigated groups showed levels of reverse torque loss, it is important to mention that no restorations became loose after a simulated clinical service of 5 years under 98 N load. While no single approach guarantees optimal stability, it is clinically significant to examine how each of these factors interacts with the mechanical properties of the screw–abutment complex. By identifying which combinations of these factors contribute to optimal stability, we enhanced implant success rates and improved patient outcomes by reducing the risks associated with screw loosening can be guaranteed.

It is important to mention that the findings of the current study are subject to certain limitations, including the number of investigated samples and the in vitro setting, which may not perfectly replicate the complexities of in vivo functional loads, bone density variations, or long-term biological responses. In the current study, prefabricated abutments were used for the investigation. However, it would be valuable to investigate screw loosening in the context of individualized CAD/CAM abutments. The customization of these abutments may influence the mechanical properties and stability of the screw connections, potentially leading to different outcomes regarding screw stability and loosening. Moreover, potential changes in screw surface condition and the state of the sealants and antimicrobial agents before and after loading are significant factors that warrant further exploration.

While the study controlled for the effects of each factor, clinical cases often involve multiple simultaneous influences that may interact differently. Examining the cumulative effect of different variables—particularly across different implant systems, various cement types, abutment angulations, and different restorations—could provide a more comprehensive and detailed understanding of implant screw stability. Future research exploring the above-mentioned aspects could provide deeper insights into optimizing implant treatment and enhancing clinical performance.

## 5. Conclusions

Within the limitations of this in vitro study, it can be concluded that screw-retained restorations are more prone to screw loosening than cement-retained ones. Abutment angulation and the presence of sealant/antimicrobial agent seem to show no significant impact on the screw stability of implant-supported single restorations under the tested conditions of this study. However, to expand these findings and enhance clinical application, future research should focus on investigating abutments with varying angulations, different cement types, as well as individually designed CAD/CAM abutments, to assess their impact on screw stability of implant-supported restorations.

## Figures and Tables

**Table 1:** The tested groups divided according to retention type, implant/abutment angulation, and the presence or absence of a sealant or disinfectant.

Group Name	Type of Retention	Implant/Abutment Angulation	Screw Chamber Filling
Cem_control	Cement-retained	straight	No
Cem_GP	Cement-retained	straight	Gutta-percha (in screw access hole above the screw
Cem_CHX	Cement-retained	straight	Chlorhexidine gel (in screw chamber below the screw)
Cem_Ang	Cement-retained	angulated (20°)	No
Screw	Screw-retained	straight	No

**Table 2:** Mean values and standard deviations of the reverse torque difference between preloading and postloading torque values in Ncm.

Tested Group	Preloading Screw-In Torque (Ncm)	Postloading Screw-In Torque (Ncm)	Reverse Torque Difference (RTD)	Removal Torque Loss (%)
Cem_control	20.14 ± 0.12	17.88 ± 2.16	2.26 ± 2.16	11.22 ± 10.79%
Cem_GP	20.12 ± 0.09	17.90 ± 1.04	2.22 ± 1.03	11.03 ± 5.12%
Cem_CHX	20.09 ± 0.10	17.18 ± 2.57	2.91 ± 2.56	14.48 ± 13.02%
Cem_Ang	20.08 ± 0.08	17.04 ± 1.91	3.04 ± 1.91	15.13 ± 9.36%
Screw	20.11 ± 0.16	15.46 ± 1.77	4.65 ± 1.76	23.12 ± 8.90%

Cem_control: cemented, straight abutments, empty; Cem_GP: cemented, straight abutments, gutta-percha; Cem_CHX: cemented, straight abutments, chlorhexidine gel; Cem_Ang: cemented, angulated abutment, empty; Screw: screwed, straight abutment, empty.

## Data Availability

The original contributions presented in this study are included in the article. Further inquiries can be directed to the corresponding author.

## Abbreviations

The following abbreviations are used in this manuscript:

Ang	Angulated
Cem	Cemented
CHX	Chlorhexidine
FDP	Fixed dental prosthesis
GP	Gutta-percha
Ncm	Newton centimeter
RTD	Reverse torque difference
RTV	Reverse torque value
SC	Single crown
